# The creatinine-to-cystatin C ratio as a prognostic risk-stratification biomarker in chronic kidney disease

**DOI:** 10.3389/fnut.2026.1766312

**Published:** 2026-03-09

**Authors:** Xiaohong Zeng, Dehui Liu, Licong Su, Meihua Wang, Guang Yang, Min Liang, Zhiqiang Peng

**Affiliations:** 1 Department of Respiratory and Critical Care Medicine, Ganzhou People's Hospital, Ganzhou Hospital-Nanfang Hospital, Southern Medical University, Ganzhou, China; 2 Department of Nephrology, Ganzhou People's Hospital, Ganzhou Hospital-Nanfang Hospital, Southern Medical University, Ganzhou, China; 3 Division of Nephrology, Nanfang Hospital, Southern Medical University, National Clinical Research Center for Kidney and Urological Diseases, Nanfang Hospital, State Key Laboratory of Multi-organ Injury Prevention and Treatment, Southern Medical University, Guangdong Provincial Key Laboratory of Renal Failure Research, Guangdong Provincial Institute of Nephrology, Guangzhou, China

**Keywords:** cardiovascular events, chronic kidney disease, creatinine/cystatin C ratio, muscle wasting, prognostic biomarker

## Abstract

**Background:**

The creatinine-to-cystatin C ratio (Cr/CysC) is a promising surrogate marker for muscle mass. While associated with adverse outcomes in various diseases, its prognostic utility for cardiorenal risks in the Chinese non-dialysis chronic kidney disease (CKD) population remains inadequately explored. This study aimed to investigate the association between the Cr/CysC ratio and the risks of cardiovascular disease (CVD) and CKD progression in a large Chinese CKD cohort.

**Methods:**

We conducted a retrospective cohort study of 16,031 non-dialysis CKD patients from the China Renal Data System (CRDS). Cox proportional hazards models were used to assess the relationship between the serum Cr/CysC ratio and the incidence of CVD and CKD progression over 28,189.68 person-years of follow-up.

**Results:**

In CKD patients, a higher serum Cr/CysC ratio was significantly associated with lower risks of both CVD events and CKD progression. Compared to the lowest quartile (Q1), patients in the highest quartile (Q4) had a 21% lower risk of CVD (HR: 0.79, 95% CI: 0.67–0.92) and a 49% lower risk of CKD progression (HR: 0.51, 95% CI: 0.46–0.58). When analyzed as a continuous variable, each unit increase in the log-transformed ratio was associated with a significantly reduced risk for both outcomes (*p* < 0.001).

**Conclusion:**

A higher serum Cr/CysC ratio is independently associated with reduced risks of CVD and CKD progression in Chinese patients with non-dialysis CKD. This ratio may serves as a useful prognostic marker for risk stratification in this population.

## Introduction

Chronic kidney disease (CKD) is a global public health care problem that affects nearly 10% of the total population worldwide (1) and 8.2% in China (2). The CKD population bears a disproportionately high burden of cardiovascular disease (CVD), which is a leading cause of morbidity and mortality. Notably, nearly half of the patients with advanced CKD experiencing CVD (3). It has been widely established that managements of traditional cardiovascular risk factors is fundamental to reducing CVD events (4, 5). However, suboptimal control of these factors and the presence of residual risks underscore the limitations of current management strategies and highlight the need to identify novel, modifiable risk factors.

Sarcopenia is highly prevalent in patients with CKD (6) and has been closely associated with various adverse clinical outcomes, including CVD, falls, loss of independence, increased hospitalization, and even mortality (7). Thus, sarcopenia is recognized as a significant risk factor for poor prognosis in CKD (8). Although tools like bioelectric impedance (BIA), dual energy X-ray absorptiometry (DXA), computed tomography (CT), and magnetic resonance imaging (MRI) can diagnose sarcopenia (9), their cost and operational constraints limit widespread clinical use. Given that creatinine production correlates with muscle mass, whereas cystatin C is largely independent of muscle metabolism (10), the serum creatinine-to-cystatin C ratio (Cr/CysC) has emerged as a promising, cost-effective surrogate marker for estimating muscle mass in patients with difference conditions (11–14).

Emerging evidence links a lower serum Cr/CysC ratio to increase a spectrum of adverse clinical outcomes, including all-cause mortality (15), poorer cognitive function (16), frailty (17), accelerated lung function decline (18), and CVD morbidity (19). While promising, the prognostic utility of the serum Cr/CysC ratio in CKD requires further validation. A cohort study by Hyun et al. demonstrated its independent association with all-cause mortality and CVD events in Korean patients with non-dialysis CKD (20). However, critical knowledge gaps remain. Firstly, existing studies have not systematically accounted for important confounding conditions such as thyroid dysfunction and extremes of body weight (obesity or underweight), which are known to independently influence serum levels of creatinine and cystatin C, thereby potentially biasing the serum Cr/CysC ratio (21). The absence of these key exclusion criteria casts uncertainty on the robustness and specificity of the previously observed associations. Secondly, the predictive value of serum Cr/CysC for major cardiorenal outcomes-particularly the progression of CKD itself-has been underexplored. Finally, and most importantly, it remains entirely unknown whether these findings can be generalized to the Chinese CKD population, which may exhibit distinct genetic, environmental, and clinical characteristics.

Therefore, this study seeks to validate the association between the serum Cr/CysC ratio and cardiorenal outcomes in a Chinese CKD cohort. By establishing this relationship, we aim to evaluate its utility for risk stratification, thereby informing more effective CVD prevention strategies for this vulnerable population.

## Materials and methods

### Data source and study participants

This retrospective cohort study used data from the China Renal Data System (CRDS), a validated nationwide population-based database encompassing over 7 million inpatients and outpatients treated at 28 urban academic hospitals from 1 January 2000 to 7 May 2023. The database’s accuracy and completeness are supported by previous publications (22, 23). The CRDS integrates standardized inpatient and outpatient clinical data, encompassing demographics, vital signs, diagnoses, prescriptions, laboratory tests, and surgical procedures. These data were consolidated from all participating centers and rigorously cleaned at the National Clinical Research Center for Kidney Disease in Guangzhou. The reliability of laboratory data is upheld as all hospitals passed the annual National External Quality Assessment. This study was performed in accordance with the Strengthening the Reporting of Observational Studies in Epidemiology (STROBE) guidelines.

**Figure 1:**
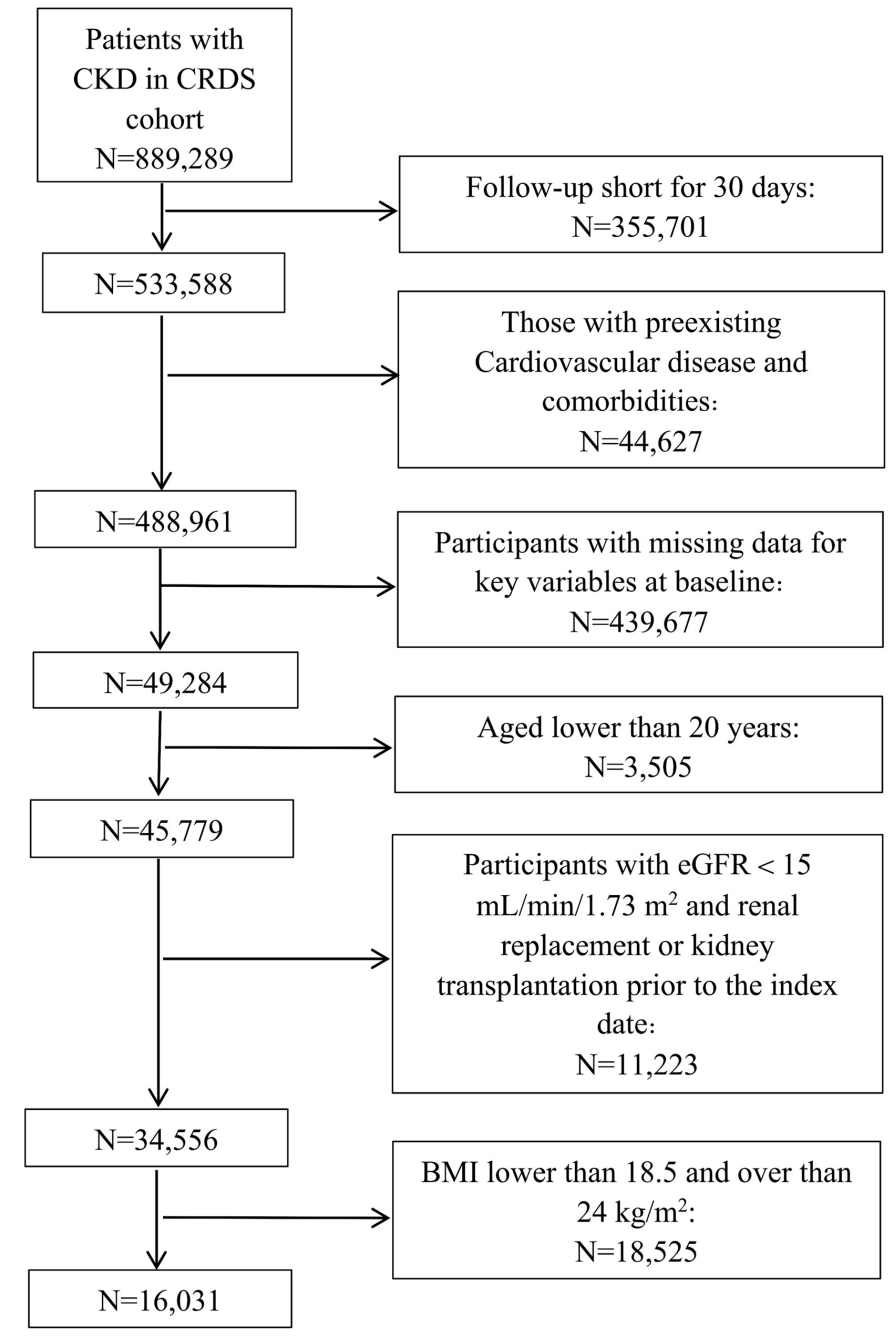
Flow chart for eligibility screening and inclusion of the study population. CKD, chronic kidney disease; CRDS, Chinese Renal Disease Data System; eGFR, estimated glomerular filtration rate; BMI, body mass index.

We included adult participants with CKD aged over 20 years. CKD was identified using the International Classification of Diseases, 10th Revision (ICD-10) code N18, or based on an eGFR <60 mL/min/1.73 m^2^, and a urinary albumin-to-creatinine ratio (UACR) ≥ 30 mg/g that were sustained for a minimum of 90 days (24). The index date was defined as the first record in the database where a participant met the CKD criteria. The exclusion criteria were as follows: (1) the absence of basic information such as age and sex at the index date, and a follow-up time that was within 30 days after the index date; (2) CVD events related to endpoint events before the index date; (3) with a history of major medical illnesses at baseline, including malignancy, liver cirrhosis, chronic obstructive pulmonary disease, hyperthyroidism, or hypothyroidism; (4) the absence of measurements of serum creatinine, serum cystatin C, weight and height data closest to the index date within the preceding 90 days; and (5) with an estimated glomerular filtration rate (eGFR) of less than 15 mL/min/1.73m^2^ or who were receiving renal replacement therapy before the index date. (6) With a body mass index (BMI) lower than 18.5 or over 24 kg/m^2^ at baseline (Figure 1). BMI was computed by dividing an individual’s body weight (in kilograms) by the square of their height (in meters squared). The eGFR was calculated using the 2021 Chronic Kidney Disease Epidemiology Collaboration (CKD-EPI) equation based on scr measurement (25), a formula that has been validated for its accuracy in Chinese populations (26).

### Assessment of exposures

The serum Cr/CysC ratio-calculated by dividing serum creatinine (mg/dL) by serum cystatin C (mg/dL)-was used as the exposure variable representing a muscle metabolic index. All laboratory parameters were assessed using values obtained during the baseline period, defined as 90 days before the index date, with the measurement closest to the index date selected when multiple values were available.

### Covariate assessment

Demographic characteristics, medical history, medication usage, and laboratory variables were extracted from the CRDS. Comorbidities, including hypertension and diabetes, were defined by ICD-10 codes (Supplementary Table 1). Charlson Comorbidity Index (CCI) was calculated by summing the scores assigned to various comorbidities (27). Prescription information was classified according to the Anatomical Therapeutic Chemical (ATC) classification system. The use of antihypertensive, antidiabetic drugs and statin drugs were identified from the 1-year period prior to the index date (Supplementary Table 2). Laboratory parameters, including serum albumin (Alb), uric acid (UA), hemoglobin (Hb), total cholesterol (TCho), low-density lipoprotein cholesterol (Ldl-c), high-density lipoprotein cholesterol (Hdl-c), and triglyceride (TG) levels, were collected closest to the index date within the preceding 90 days. Missing data (Supplementary Table 3) were assumed to be missing at random and were addressed by multiple imputation using chained equations (MICE) with 5 imputations in R (v4.3; ‘mice’ package).

### Ascertainment of outcomes

The primary outcomes were incident CVD events (28), including atherosclerotic cardiovascular disease, heart failure, stroke, atrial fibrillation, and peripheral artery disease. Details of definition of these outcomes were described in Supplementary Table 1. The secondary outcome was CKD progression, assessed by follow-up laboratory test results in the CRDS, defined as any of the following: a ≥ 40% decrease in the eGFR from baseline, a twofold increase in the serum creatinine level from baseline, an eGFR <15 mL/min/1.73 m^2^, initiation of maintenance hemodialysis/peritoneal dialysis, or a kidney transplant event (29–31). Person-years of follow-up for each participant were calculated from the date of the first diagnosis of CKD to the date of either the occurrence of an event of interest or the date of their last medical record, whichever came first.

### Statistical analysis

Continuous data are shown as the means (SDs) for normally distributed data and medians (IQRs) for nonnormally distributed data, and categorical data are expressed as counts and percentages. We categorized the serum Cr/CysC ratio into quartiles. Differences in the baseline characteristics according to the quartiles of the serum Cr/CysC ratio were examined using one-way analysis of variance, the Kruskal-Walli’s test, and the chi-square test, as appropriate. The associations between these quartiles and the risks of CVD events and CKD progression were assessed using multivariable-adjusted Cox proportional hazards regression models, with the lowest quartile (Q1) serving as the reference group. The proportional hazards assumption was verified using Schoenfeld residuals, with no significant violations detected. To this end, a Cox model was constructed for the total population and fully adjusted for the following covariates for age, sex, CCI, Alb, Hb, TG, Ldl-c, Hdl-c, TCho, UA, eGFR (all continuous), and use of antihypertensive, antidiabetic, and statin drugs. The distribution of serum Cr/CysC ratio was markedly skewed (Supplementary Figure 1). To satisfy the linearity assumption for continuous variables in Cox proportional hazards regression and to better characterize its association with clinical outcomes, the natural logarithm of serum Cr/CysC ratio was used in the primary regression models. We further assessed the association of both the original and the log-transformed serum Cr/CysC ratio with the outcomes using restricted cubic spline (RCS) analyses to explore potential non-linearity. Additionally, subgroup analyses were performed by age (<65, ≥65 years), sex, eGFR (<45, ≥45 mL/min/1.73 m^2^), and medication use, with interaction terms tested within the fully adjusted model. To assess the robustness of our findings, we performed several sensitivity analyses: (1) excluding participants who developed outcomes within the first year to minimize reverse causality; (2) repeating analyses using a complete-case dataset without imputation; and (3) applying competing risk models, treating all-cause death as a competing event. Finally, we calculated the E value to mitigate potential bias arising from unmeasured confounders.

To assess the incremental predictive value of the log-transformed Cr/CysC ratio, the C statistic for existing baseline risk models (adjusted for age; sex; CCI, UA, TCho, Alb, eGFR, Hdl-c, Ldl-c, TG, and Hb values and the use of antidiabetic agents, antihypertensive agents, and statin agents). Subsequently, the C statistic for the addition of the log-transformed serum Cr/CysC ratio to existing models was assessed to evaluate the incremental value. Net reclassification improvement and integrated discrimination improvement indexes were also assessed to determine reclassification and discrimination (32). Bootstrap estimation was performed to calculate 95% CIs of the net reclassification improvement.

All analyses were conducted using R version 4.3.1.^1^ All reported *p* values were 2-sided. A cutoff of 0.05 was considered to indicate statistical significance.

## Results

### Baseline characteristics of the study population

A total of 16,031 CKD patients were enrolled in the study. Based on the serum Cr/CysC ratio, patients were categorized into quartiles as follows: 4,008 (25.0%) in Quartile 1 (Q1), 4,008 (25.0%) in Q2, 4,007 (25.0%) in Q3, and 4,008 (25.0%) in Q4. The baseline characteristics of the study participants are presented according to the quartiles of serum Cr/CysC ratio (Table 1). The median age was 55 years (IQR: 41–66 years), and 9,094 patients were male (56.7%). Baseline characteristics of those excluded from the analysis due to BMI lower than 18.5 or over 24 kg/m^2^ were similar to those who were included in the analysis (Supplementary Table 4). Compared with participants in quartile 1, those in the higher quartiles were more likely to be younger men; have lower Ldl-c, TCho, eGFR, TG levels; have higher Hb, Hdl-c, and Alb levels; and not have a history of hypertension or diabetes (Table 1).

**Table 1:** Baseline characteristics according to the quartiles of serum Cr/CysC ratio in 16,031 adults with chronic kidney disease.

Characteristics	Overall, *n* (%)	Quartiles of serum Cr/CysC ratio				*p*-values
		Q1	Q2	Q3	Q4	
Total	16,031	4,008	4,008	4,007	4,008	
Age, median (IQR), yr.	55 [41, 66]	57 [44, 68]	57 [45, 67]	55 [41, 65]	52 [37, 63]	*p* < 0.01
Male	9,094 (56.7)	1,612 (40.2)	2074 (51.7)	2,509 (62.6)	2,899 (72.3)	*p* < 0.01
Laboratory test indicators, median (IQR)						*p* < 0.01
Scr (mg/dL)	0.96 [0.74, 1.35]	0.77 [0.61, 1.04]	0.90 [0.72, 1.23]	1.03 [0.80, 1.44]	1.21 [0.92, 1.70]	*p* < 0.01
Cysc (mg/dL)	1.16 [0.90, 1.61]	1.34 [1.06, 1.83]	1.17 [0.94, 1.59]	1.11 [0.87, 1.55]	0.99 [0.74, 1.41]	*p* < 0.01
Cr/CysC	0.84 [0.70, 1.02]	0.60 [0.53, 0.65]	0.78 [0.74, 0.81]	0.92 [0.88, 0.97]	1.17 [1.08, 1.33]	
eGFR (ml/min/1.73m^2^)	80[52,101]	93 [66, 1,091]	83 [56, 102]	75 [49, 98]	63 [42, 93]	*p* < 0.01
UA (mmol/L)	347 [272, 433]	333 [261, 419]	339[271, 419]	353 [278, 437]	363 [283, 456]	*p* < 0.01
Alb (g/L)	37.50 [32.10, 41.60]	34.30 [28.30, 39.10]	37.60 [32.36, 41.60]	38.30 [33.60, 42.20]	39.20 [34.50, 43.00]	*p* < 0.01
Ldl -c(mmol/L)	2.57 [1.94, 3.34]	2.53 [1.84, 3.44]	2.63 [1.99, 3.40]	2.59 [1.99, 3.31]	2.52 [1.93, 3.23]	*p* < 0.01
Tcho (mmol/L)	4.35 [3.55, 5.37]	4.34 [3.40, 5.55]	4.45 [3.63, 5.46]	4.34 [3.62, 5.33]	4.28 [3.54, 5.18]	*p* < 0.01
Hdl-c(mmol/L)	1.10 [0.88, 1.38]	1.05 [0.81, 1.36]	1.12 [0.90, 1.40]	1.11 [0.89, 1.38]	1.11 [0.90, 1.37]	*p* < 0.01
Hb (g/L)	123 [107, 138]	115 [99, 131]	123[109, 137]	126 [110, 140]	129 [112, 143]	*p* < 0.01
TG (mmol/L)	1.31 [0.94, 1.92]	1.46 [1.03, 2.13]	1.31 [0.94, 1.93]	1.26 [0.90, 1.79]	1.25 [0.92, 1.82]	*p* < 0.01
Vital signs, median (IQR)						*p* < 0.01
SBP(mmHg)	121 [108, 135]	120 [110, 136]	121[109, 136]	121 [109, 135]	120. [105, 135]	*p* < 0.01
DBP (mmHg)	76 [70, 84]	76 [69, 83]	76 [69, 84]	76 [69, 84]	77 [70, 86]	*p* < 0.01
BMI (kg/m^2^)	21.80 [20.45, 22.94]	21.64 [20.34, 22.83]	21.80 [20.49, 22.96]	21.87 [20.43, 22.99]	21.97 [20.57, 23.05]	*p* < 0.01
Comorbidities						*p* < 0.01
Hypertension	2,686 (16.8)	765 (18.4)	688 (17.5)	737 (17.0)	496 (13.8)	*p* < 0.01
Diabetes	1870 (11.7)	560 (13.5)	538 (13.7)	466 (10.7)	306 (8.5)	*p* < 0.01
CCI	1 [0, 3]	2 [0, 3]	2 [0, 3]	1 [0, 2]	1 [0, 2]	*p* < 0.01
Antidiabetic agents	2,275(14.2)	653 (16.3)	583 (14.5)	499 (12.5)	540 (13.5)	*p* < 0.01
Antihypertension agents	3,844 (24.0)	1,009 (25.2)	966 (24.1)	935 (23.3)	934 (23.3)	*p* < 0.01
Statins agents	1,691 (10.5)	422 (10.5)	438 (10.9)	442 (11.0)	389 (9.7)	*p* < 0.01

BMI, body mass index; Scr, serum creatinine; Cysc, Cystatin C; Cr/CysC, Creatinine-to-Cystatin C ratio; eGFR, estimated glomerular filtration rate; Hdl-c, high-density lipoprotein cholesterol; Tcho, total cholesterol; Ldl-c, low-density lipoprotein cholesterol; TG, triglyceride; SBP, systolic blood pressure. DBP, diastolic blood pressure; Alb, Albumin; Hb, hemoglobin; UA, uric acid; CCI, Charlson Comorbidity Index.

### Associations of serum Cr/CysC ratio with CVD incidence and CKD progression

**Figure 2:**
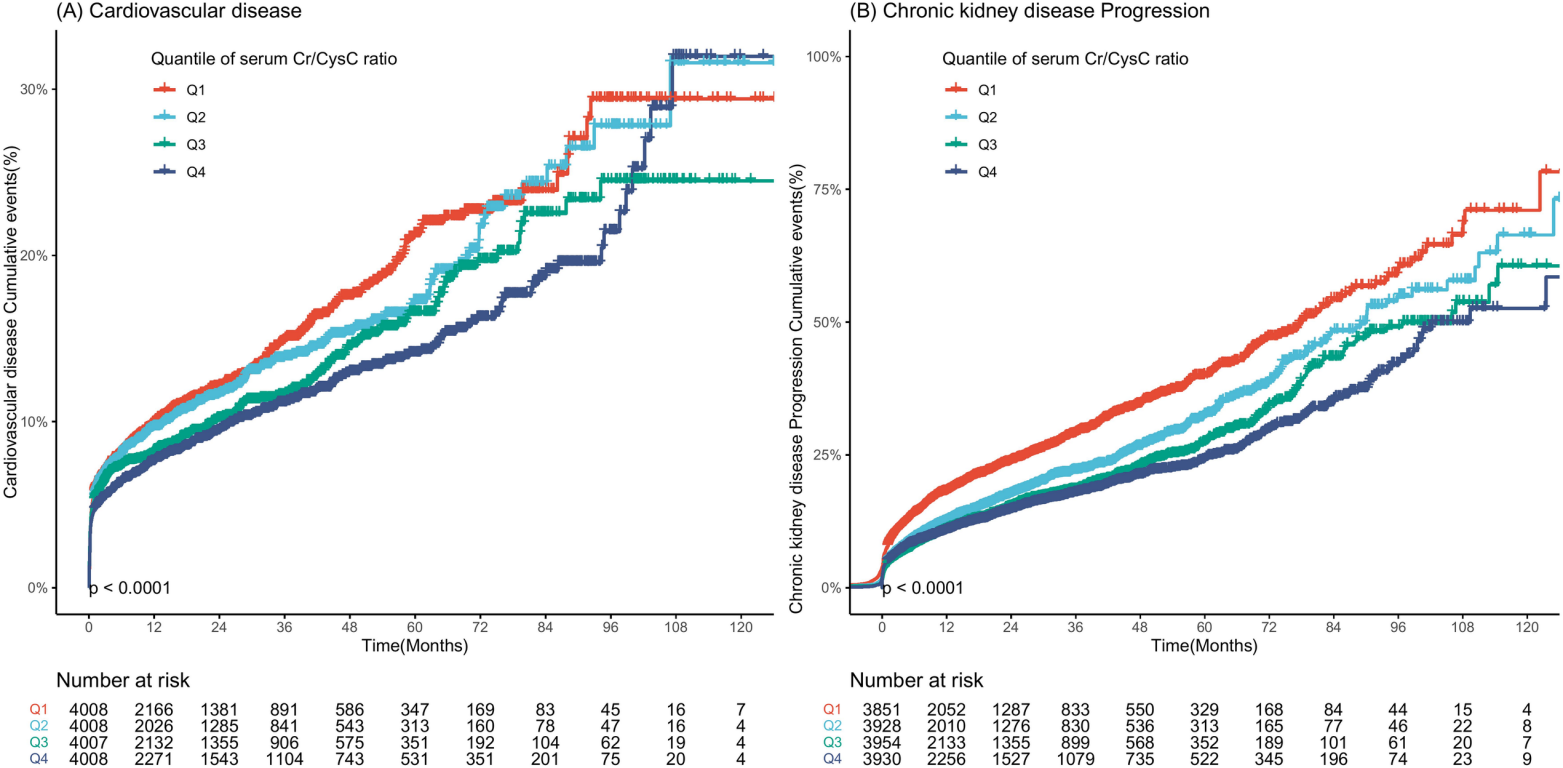
Time-to-event distribution for **(A)** CVD events and **(B)** CKD progression, stratified by quartiles of the serum Cr/CysC ratio.

Across 28189.68 person-years of observation (median follow-up of 474 days, IQR:168–1,092 days), 1,759 (11.0%) participants experienced CVD events, and 3,000 (18.7%) participants experienced CKD progression. The time–to-event distributions are displayed in Figures 2A,B. Individuals in Quartile 1 were less likely to develop CVD and CKD progression than those with in Quartile 4. Table 2 shows the Cox regression models used to examine the associations between serum Cr/CysC and CVD risk and CKD progression. Compared with participants in quartile 1, those in quartile 4 presented significantly lower risks of CVD [Quartile 2, HR: 0.87 (95% CI: 0.76–0.99); Quartile 3, HR: 0.80 (95% CI: 0.69–0.92); and Quartile 4, HR: 0.79 (95% CI: 0.67–0.92)] and CKD progression [Quartile 2, HR: 0.71 (95% CI: 0.64–0.78); Quartile 3, HR: 0.57 (95% CI: 0.51–0.64); Quartile 4, HR: 0.51 (95% CI: 0.46–0.58)]. This inverse trend persisted when serum Cr/CysC and the log-transformed Cr/CysC ratio was analyzed as a continuous variable, with each unit increase associated with a significantly lower risk of CVD events and CKD progression (both *p* < 0.001).

**Table 2:** Cox regression models of the association between serum Cr/CysC ratio and CVD events and CKD progression.

Variables	Cases/person years	Model 1		Model 2		Model 3	
		HR (95% CI)	*p* value	HR (95% CI)	*p* value	HR (95% CI)	*p* value
Cardiovascular disease							
Q1	496/6585.2	Reference		Reference		Reference	
Q2	454/6562.3	0.93(0.82–1.06)	0.32	0.92(0.81–1.05)	0.24	0.87(0.76–0.99)	< 0.05^*^
Q3	412/6980.4	0.83(0.72–0.94)	< 0.01^**^	0.89(0.77–1.01)	0.08	0.80(0.69–0.92)	< 0.01^**^
Q4	397/8061.8	0.74(0.65–0.84)	< 0.001^***^	0.87(0.76–0.99)	< 0.05^*^	0.79(0.67–0.92)	< 0.01^**^
As continuous variable							
Cr/CysC		0.65(0.54–0.77)	< 0.001^***^	0.78(0.65–0.94)	< 0.01^**^	0.71(0.57–0.87)	< 0.001^***^
LogCr/CysC		0.44(0.31–0.61)	< 0.001^***^	0.58(0.41–0.85)	< 0.01^**^	0.43(0.28–0.66)	< 0.001^***^
Chronic kidney disease progression							
Q1	993/6585.2	Reference		Reference		Reference	
Q2	7171/6562.3	0.71(0.65–0.79)	< 0.001^***^	0.68(0.62–0.75)	< 0.001^***^	0.71(0.64–0.78)	< 0.001^***^
Q3	634/6980.4	0.61(0.54–0.66)	< 0.001^***^	0.57(0.51–0.63)	< 0.001^***^	0.57(0.51–0.64)	< 0.001^***^
Q4	656/8061.8	0.56(0.51–0.62)	< 0.001^***^	0.53(0.48–0.59)	< 0.001^***^	0.51(0.46–0.58)	< 0.001^***^
As continuous variable							
Cr/CysC		0.54(0.47–0.62)	< 0.001^***^	0.51(0.43–0.58)	< 0.001^***^	0.51(0.43–0.59)	< 0.001^***^
LogCr/CysC		0.19(0.15–0.25)	< 0.001^***^	0.15 (0.12–0.20)	< 0.01^**^	0.13(0.09–0.18)	< 0.001^***^

Model 1: crude model.

Model 2: adjusted for age, sex.

Model 3: adjusted for adjusted for age; sex; CCI, UA, TCho, Alb, eGFR, Hld-c, TG, Ldl-c, and Hb values; and the use of antidiabetic agents, antihypertensive agents, and statin agents. Asterisks indicate significance levels (^*^p < 0.05, ^**^p < 0.01, ^***^p < 0.001).

Multiple sensitivity analyses confirmed the robustness of our primary findings. The association between the quartiles of serum Cr/CysC ratio and cardiorenal outcomes remained consistent when using a competing risk model, after excluding early-onset events to mitigate reverse causality, and in a complete-case analysis (Supplementary Table 5). The high E-values (1.8 for CVD events and 3.3 for CKD progression) further indicated that the results are robust to potential unmeasured confounding.

### Shapes of the associations between serum Cr/CysC ratio and outcomes

**Figure 3:**
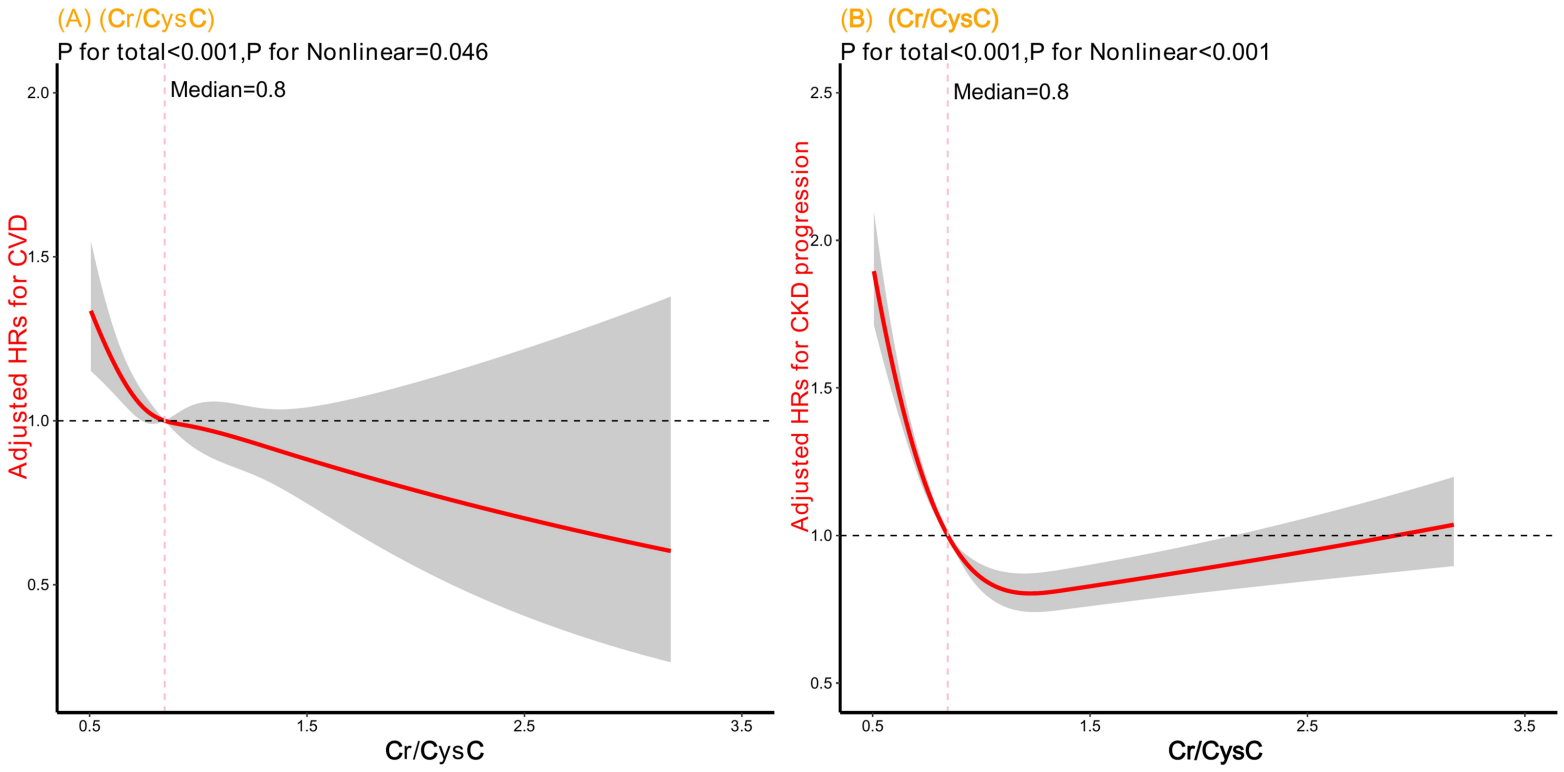
Cubic spline model shows relationship of serum Cr/CysC ratio with **(A)** CVD events and **(B)** CKD progression. Model adjusted for age; sex; CCI, UA, TCho, Alb, eGFR, Hdl-c, TG, Ldl-c, and Hb values; and the use of antidiabetic agents, antihypertensive agents, and statin agents.

We used restricted cubic splines to determine the associations of the serum Cr/CysC ratio with cardiorenal outcomes among patients with CKD. Figure 3 depicts the nonlinear associations of the serum Cr/CysC ratio with adverse outcomes in patients with CKD; Figure 3 A shows its relationship with the risk of CVD events (*P* for nonlinear = 0.046), and Figure 3B shows its relationship with the risk of CKD progression (*P* for nonlinear<0.001). The associations of the log-transformed serum Cr/CysC ratio with cardiorenal outcomes were depicted in Supplementary Figure 2. However, wide confidence intervals did not allow us to draw reliable conclusions with enough confidence.

### Subgroup analyses

**Figure 4:**
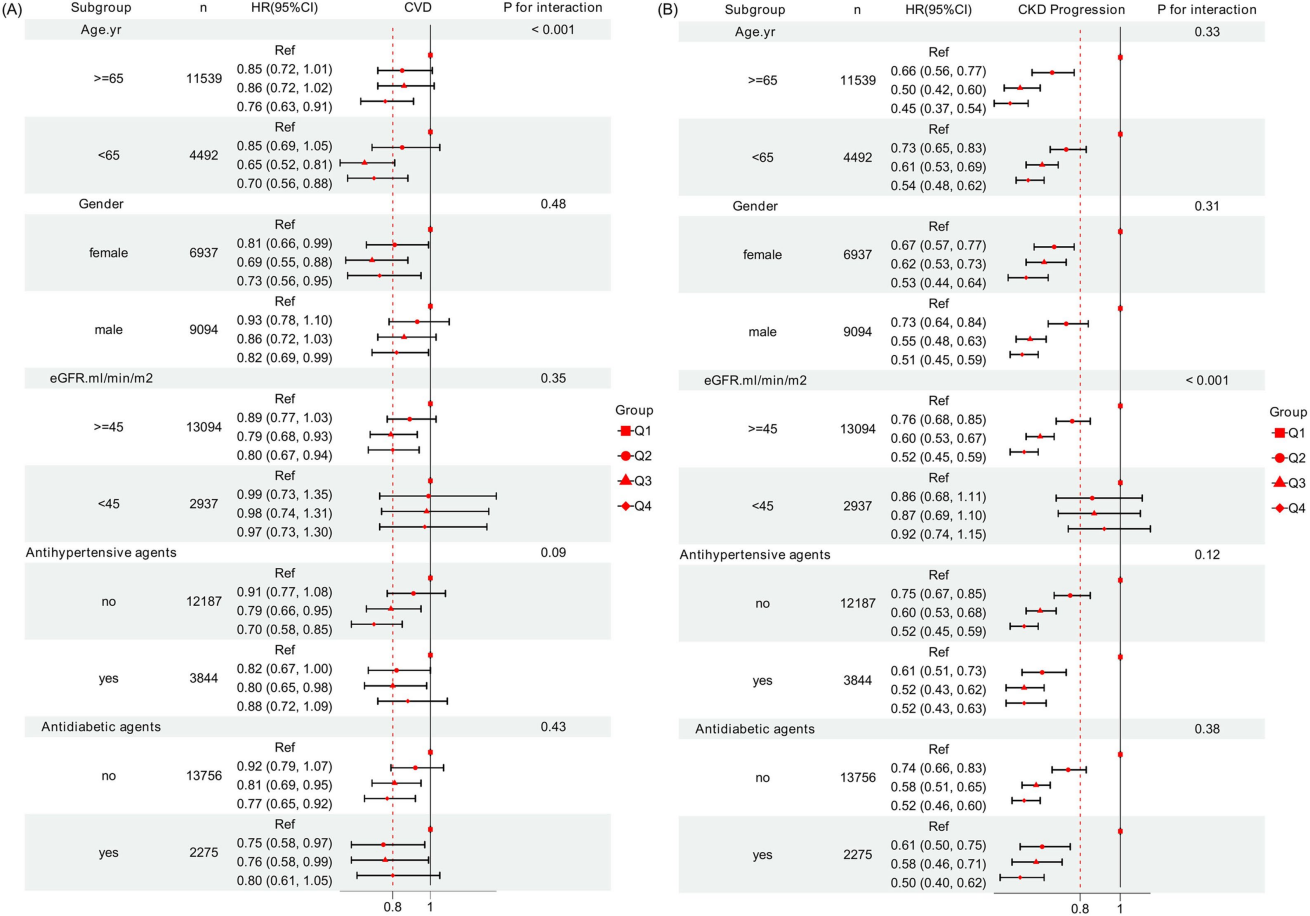
Subgroup analysis of the associations between serum Cr/CysC ratio with **(A)** CVD events and **(B)** CKD progression among patients with CKD. Model adjusted for age; sex; CCI, UA, TCho, Alb, eGFR, HDL-c, TG, LDL-c, and Hb values; and the use of antidiabetic agents, antihypertensive agents, and statin agents.

Subgroup analysis revealed a significant interaction by age (*P* for interaction < 0.001, Figure 4). The reduction in CVD risk associated with the highest quartile (Q4) of serum Cr/CysC ratio was more pronounced in patients aged <65 years compared to those aged ≥65 years. The association of serum Cr/CysC ratio quartiles with CKD progression was modified by eGFR (*P* for interaction < 0.001), with a more substantial risk reduction in Q4 observed among those with higher baseline kidney function.

### Log-transformed serum Cr/CysC ratio and cardiovascular disease and chronic kidney disease progression risk reclassification and discrimination

To evaluate incremental value, C statistics was assessed when the log-transformed serum Cr/CysC ratio was added to existing baseline risk models. The C statistic for baseline risk models was 0.809 (95% CI, 0.799–0.818) for CVD events and 0.689 (95% CI, 0.679–0.698) for CKD progression. The addition of log-transformed serum Cr/CysC ratio to this model improved the C index by 0.001 for CVD events and 0.008 for CKD progression. Reclassification calibration assessments revealed that the continuous net reclassification improvements for 1 year CVD events and CKD progression were 0.014 (95% CI, −0.011-0.026) and 0.023 (95% CI, 0.008–0.053), respectively. The integrated discrimination improvements for 1 year CVD events and CKD progression were 0.001 (95% CI, 0.0001–0.002) and 0.008 (95% CI, 0.005–0.011), respectively (Supplementary Table 6).

## Discussion

We found that higher serum Cr/CysC ratio was strongly associated with a lower risk of CVD events and CKD progression when adjusted the demographics, comorbidities, and clinical factors at baseline. Our findings not only align with the Korean KNOW-CKD cohort (20), strengthening the evidence for the serum Cr/CysC ratio as a universal prognostic marker in CKD, but also provide more robust support for its specificity and reliability through methodological refinements-specifically, by being the first to exclude key confounding factors in a Chinese population.

The inverse association between the serum Cr/CysC ratio and cardiorenal risk is consistent with the ratio’s function as a surrogate marker for skeletal muscle mass. This interpretation is supported by both the baseline profile of high-ratio participants (characterized by younger age, male sex, and fewer comorbidities) in our findings and prior epidemiological evidence (19). Given its established associations with diverse clinical outcomes across multiple populations, its prognostic relevance appears to transcend specific disease entities, geographic regions, and comorbid conditions. To further solidify its generalizability, future studies are warranted to validate this association specifically in Western CKD populations.

Subgroup analysis revealed that the associations of serum Cr/CysC ratio with CVD risk and CKD progression were differentially modified by age and baseline kidney function. The more pronounced cardioprotective effect in younger patients can be attributed to their fewer comorbidities, which makes muscle mass a cleaner biomarker of physiologic reserve (33), making the ratio a clearer marker of physiologic reserve against cardiovascular risk. Conversely, the benefit for slowing CKD progression was stronger in patients with higher baseline eGFR. This suggests that in the context of early-stage CKD adults with declining physiologic reserve, the ratio-reflecting muscle and nutritional status-becomes a critical indicator of physiologic resilience against disease progression (34). In summary, the clinical utility of the serum Cr/CysC ratio as a composite biomarker is population specific.

The population-specific utility highlighted above is further clarified and strengthened by the methodological refinements of our study. Specifically, by systematically excluding individuals with thyroid dysfunction or abnormal body mass, which were two important non-muscular determinants of serum creatinine and cystatin C (21, 35), we substantially reduce the potential for confounding. This approach strengthens the inference that the observed association between a low serum Cr/CysC ratio and increased risk of cardiorenal outcomes is more specifically tied to skeletal muscle mass rather than other metabolic or inflammatory conditions. Consequently, our findings not only validate previous reports but also extend them by offering a more rigorously contextualized and etiologically relevant understanding of the serum Cr/CysC ratio as a marker of muscle-related health in CKD.

The potential pathophysiological mechanisms that may underlie the association between serum Cr/CysC ratio and CVD events and CKD progression in patients with CKD are discussed below. First, patients with CKD had an increased risks of muscle wasting, such as poor appetite, inflammation, insulin resistance, and metabolic acidosis (36). Sarcopenia diminishes the body’s metabolic reservoir and reduces its capacity for glucose disposal, which exacerbates insulin resistance and metabolic dysregulation (37). This insulin resistance, in turn, activates pathways such as protein kinase C and nuclear factor-κB, leading to excessive production of reactive oxygen species (ROS) (38). The resulting oxidative stress triggers endothelial dysfunction and systemic inflammation (39), key drivers of both cardiovascular disease and CKD progression. Moreover, hyperinsulinemia can further inhibit nitric oxide release, promoting vascular fibrosis and atherosclerosis (39). Second, beyond its role in glucose metabolism, skeletal muscle also functions as an endocrine organ. Muscle loss leads to reduced synthesis and secretion of myokines-protective factors such as apelin, follistatin-like 1, and irisin. Apelin improves insulin sensitivity, stimulates angiogenesis, promotes vasodilation, and enhances myocardial contractility (40); follistatin-like 1 enhances endothelial function and supports vascular regeneration (41); and irisin improves mitochondrial function, promotes myocardial regeneration and angiogenesis, and reduces fibrosis (42). The decline in these myogenic factors directly impairs cardiovascular repair mechanisms, vascular health, and metabolic homeostasis, thereby synergistically accelerating vascular and renal damage in the context of chronic inflammation. Finally, diminished muscle mass and strength directly contribute to physical frailty and reduced functional capacity, which are themselves powerful predictors of increased morbidity and mortality in chronic disease states. Thus, the serum Cr/CysC ratio likely encapsulates a composite risk profile, integrating metabolic, inflammatory, and functional decline, all of which converge to worsen prognosis in CKD patients. However, the exact mechanisms of the relationship between serum Cr/CysC and cardiorenal outcomes remains to be elucidated by future biological studies.

Our findings carry direct implications for clinical practice. The serum Cr/CysC ratio, which is derived from routine laboratory tests, offers a pragmatic and cost-effective tool for risk stratification in CKD patients. A low ratio could serve as a practical indicator to identify individuals with probable sarcopenia and high cardiorenal risk, helping to detect at-risk patients who could be overlooked in routine assessments. This identification should prompt a multidisciplinary evaluation, leading to targeted interventions such as personalized nutritional support and tailored physical activity regimens. Future prospective studies are warranted to establish specific cutoff values for the serum Cr/CysC ratio in different CKD populations and to determine whether interventions aimed at improving this ratio-through, for example, resistance exercise or nutritional supplementation-can effectively reduce the incidence of cardiovascular events and halt CKD progression.

Several limitations should be acknowledged. First, using multiple imputation methods to handle missing data may introduce bias into the observed relationships. To mitigate this bias, we conducted a sensitivity analysis, and the results remained consistent when we reanalyzed the data using only complete cases, excluding missing values. Second, although we adjusted our models for many covariates, several residual confounding such as protein intake, volume status, exercise habits, inflammatory markers, UACR cannot be entirely ruled out, which is a common limitation in observational studies. Third, as this is an observational study, nonrespondents in subsequent surveys may introduce selection bias. Fourth, we did not measure muscle mass using an image analysis or muscle function by grip strength or walking speed as the nature of our data source. Fifth, the serum Cr/CysC ratio in our analysis was based on a single measurement, which cannot capture its potential temporal variation over time. Future studies incorporating longitudinal measurements of this ratio are warranted to explore the association between its dynamic changes and clinical outcomes. Finally, our study participants were all Chinese patients with CKD. Therefore, it might be difficult to generalize our findings to all patients with CKD. To extrapolate our findings, further studies evaluating muscle mass-targeted interventions are needed.

## Conclusion

In conclusion, the serum Cr/CysC ratio is independently associated with an increased risk of CVD events and CKD progression among adults with non-dialysis CKD. Given that this ratio serves as an accessible surrogate biomarker for sarcopenia, it could be served as a novel prognostic tool for risk stratification in CKD patient management. Prospective studies are warranted to confirm these findings in broader populations.

## Data Availability

The original contributions presented in the study are included in the article/Supplementary material, further inquiries can be directed to the corresponding authors.

## Footnotes

1
http://www.R-project.org
