# Supplementary material for: The creatinine-to-cystatin C ratio as a prognostic risk-stratification biomarker in chronic kidney disease
Source: Front Nutr. 2026 Mar 9;13:1766312. doi: 10.3389/fnut.2026.1766312 (PMC13006245; doi:10.3389/fnut.2026.1766312)
Supplement: Supplementary file 1 [file Table_1.doc]

**The Creatinine-to-Cystatin C ratio as a prognostic risk-stratification biomarker in chronic kidney disease**

Xiaohong Zeng^1†^, Dehui Liu^2†^, Licong Su^3†^,Meihua Wang^2^, Guang Yang^2^, Min Liang^3*^ and Zhiqiang Peng^2*^

**Supplementary material content**

**Table 1.** Definition of the outcome measurements.

**Table 2.** Anatomical therapeutic chemical (ATC) codes used to identify pharmaceutical therapies the participants received.

**Table 3.** Proportion of missing data for covariates.

**Table 4.** Baseline characteristics of the study participants stratified by BMI eligibility criteria.

**Table 5.** Sensitivity analysis for the association between serum Cr/Cys ratio and CVD and CKD progression.

**Table 6.** C-statistic, net reclassification improvement (NRI), and integrated discrimination improvement (IDI) for 1-year CVD and CKD progression after adding the log-transformed serum Cr/CysC ratio to the basic model.

**Figure 1.** Box plots showing the distribution of the serum Cr/Cys ratio before and after log-transformation.

**Figure 2.** Cubic spline model shows relationship of log-transformed serum Cr/CysC ratio with outcomes.

**Table 1.** Definition of the outcome measurements.

| **Outcomes** | **Definition and ICD-10 codes** |
| --- | --- |
| Prior cardiovascular disease (CVD) | Prior CVD was defined as having the any of the following diseases diagnosed prior to baseline: atherosclerotic cardiovascular disease , heart failure (HF), atrial fibrillation (AF), peripheral arterial disease (PAD) or stroke. |
| Atherosclerotic cardiovascular disease (ASCVD) | ASCVD was defined using the ICD-10 codes of I21.0|I21.1|I21.2|I21.3|I21.4|I21.9|I25.2. The event was defined as the first occurrence of the ICD codes above in any follow-up visits after the index day. |
| Hospitalization for heart failure (HF) | Hospitalization for HF was defined as the first occurrence of the diagnosis using the ICD-10 code I50.0|I50.101|I50.103|I50.104|I50.907 as the major diagnosis at any hospitalized follow-up visit after the index day. |
| Atrial fibrillation (AF) | AF was defined as the first occurrence of the diagnosis using the ICD-10 code I48 as the major diagnosis at any hospitalized follow-up visit after the index day. |
| Peripheral arterial disease (PAD) | PAD was defined as the first occurrence of the diagnosis using the ICD-10 code I74.2|I74.3|I74.4|I74.5 as the major diagnosis at any hospitalized follow-up visit after the index day. |
| Stroke | Stroke was defined as the first occurrence of the diagnosis using the ICD-10 code I60|I61|I63|I69 as the major diagnosis at any hospitalized follow-up visit after the index day. |
| **Comorbidities** | **ICD-10 codes** |
| Hypertension | I10|I11|I12|I13 |
| Diabetes | E10|E11|E12|E13|E14 |
| Tumor | C00-C97 |
| Hepatic cirrhosis | K70|K74|B18|I85|I86|K72|K76 |
| Chronic obstructive pulmonary disease (COPD) | J44 |
| Thyroid disease | E03|E05 |

**Table 2. Anatomical therapeutic chemical (ATC) codes used to identify pharmaceutical therapies the participants received.**

| **Drugs** | **ATC code** |
| --- | --- |
| β blocking agents | C07AA|C07AB|C07AG|C07BA|C07BB|C07BG|C07CA|C07CB|C07CG|C07DA|C07DB|C07EA|C07EB|C07FB|C07FX |
| Calcium channel blockers | C08CA|C08CX|C08DA|C08DB|C08EAC08EX|C08GA |
| ACEI | C09A, C09B |
| ARB | C09C, C09D |
| Statins | C10AA |
| Antidiabetic drugs | A10BX02, 03, 08, A10BH, A10BG, A10BB, A10BF, A10BA02 |

Abbreviations:ACEI: angiotensin-converting enzyme inhibitor; ARB: angiotensin II receptor blocker.

Table 3. Proportion of missing data for covariates.

| **Variant** | **Proportion (%)** |
| --- | --- |
| Sex | 0 |
| Age | 0 |
| BMI | 0 |
| SBP | 0 |
| DBP | 0 |
| Hypertension | 0 |
| Diabetes | 0 |
| Scr | 0 |
| Cysc | 0 |
| eGFR | 0 |
| Alb | 0.63 |
| Hb | 0.68 |
| Tcho | 9.4 |
| TG | 11.1 |
| Hdl-c | 11.8 |
| Ldl-c | 11.9 |
| UA | 13.1 |

Abbreviations: BMI, body mass index; eGFR, estimated glomerular filtration rate; Hdl-c, high-density lipoprotein cholesterol; Tcho, total cholesterol; Ldl-c, low-density lipoprotein cholesterol; TG, triglyceride; SBP, systolic blood pressure. DBP, diastolic blood pressure; Alb, Albumin; Hb, hemoglobin; UA, uric acid; Scr, serum creatinine;Cysc, Cystatin C.

**Table 4.** Baseline characteristics of the study participants stratified by BMI eligibility criteria.

| Characteristics | Excluded | Included |
| --- | --- | --- |
| Total | 18525 | 16031 |
| Age, median (IQR), yr. | 55.00 [43.00, 65.00] | 55.00 [41.00, 66.00] |
| Male | 11824 (63.8) | 9094 (56.7) |
| Laboratory test indicators, median (IQR) |  |  |
| Scr (mg/dl) | 1.02 [0.77, 1.38] | 0.96 [0.74, 1.35] |
| Cysc (mg/dl) | 1.17 [0.91, 1.58] | 1.16 [0.90, 1.61] |
| Cr/CysC | 0.88 [0.72, 1.05] | 0.84 [0.70, 1.02] |
| eGFR (ml/min/1.73m^2^) | 76.49[51.57,99.37] | 79.62[52.43,101.24] |
| UA (mmol/L) | 377.00 [298.00, 463.00] | 347.00 [272.00, 433.00] |
| Alb (g/L) | 38.50 [33.20, 42.50] | 37.50 [32.10, 41.60] |
| Ldl -c(mmol/L) | 2.66 [2.03, 3.45] | 2.57 [1.94, 3.34] |
| Tcho (mmol/L) | 4.45 [3.64, 5.47] | 4.35 [3.55, 5.37] |
| Hdl-c(mmol/L) | 1.02 [0.83, 1.26] | 1.10 [0.88, 1.38] |
| Hb (g/L) | 132.00 [116.00, 147.00] | 123.00 [107.00, 138.00] |
| TG (mmol/L) | 1.64 [1.13, 2.45] | 1.31 [0.94, 1.92] |
| Vital signs, median (IQR) |  |  |
| SBP (mmHg) | 125.00 [110.00, 140.00] | 121.00 [108.00, 135.00] |
| DBP (mmHg) | 80.00 [70.00, 88.00] | 76.00 [70.00, 84.00] |
| BMI (kg/m^2^) | 26.11 [24.68, 28.09] | 21.80 [20.45, 22.94] |
| Comorbidities |  |  |
| Hypertension | 4708 (25.4) | 2686 (16.8) |
| Diabetes | 3041 (16.4) | 1870 (11.7) |
| CCI | 1.00 [0.00, 3.00] | 1.00 [0.00, 3.00] |
| Antidiabetic agents | 3325(17.9) | 2275(14.2) |
| Antihypertension agents | 6082 (32.8) | 3844 (24.0) |
| Statins agents | 2968 (16.0) | 1691 (10.5) |

Notes:Participants were stratified by BMI eligibility criteria for the primary analysis (Included: 18.5 < BMI < 24.0 kg/m²; Excluded: BMI ≤ 18.5 or ≥ 24.0 kg/m²).

Abbreviations: BMI, body mass index; Scr, serum creatinine; Cysc, Cystatin C; Cr/CysC, Creatinine-to-Cystatin C ratio; eGFR, estimated glomerular filtration rate; Hdl-c, high-density lipoprotein cholesterol; Tcho, total cholesterol; Ldl-c, low-density lipoprotein cholesterol; TG, triglyceride; SBP, systolic blood pressure. DBP, diastolic blood pressure; Alb, Albumin; Hb, hemoglobin; UA, uric acid; CCI, Charlson Comorbidity Index.

**Table 5.** Sensitivity analysis for the association between serum Cr/Cys ratio and CVD and CKD progression

|  | **Quartiles of serum Cr/Cys C ratio ^a^** | | | |
| --- | --- | --- | --- | --- |
|  | **Q1** | **Q2** | **Q3** | **Q4** |
| **Cardiovascular Disease** |  |  |  |  |
| Excluding patients with less than 1-year of follow-up (N=8,497) | Reference | 0.73(0.56-0.96) | 0.74(0.56-0.98) | 0.72(0.54-0.96) |
| Using the complete dataset without imputation (N=12,043) | Reference | 0.85(0.73-1.00) | 0.73 (0.61-0.87) | 0.74 (0.62-0.89) |
| Competing risk model | Reference | 0.87(0.75-1.00) | 0.82(0.71-0.95) | 0.80(0.68-0.94) |
| **Chronic kidney disease Progression** |  |  |  |  |
| Excluding patients with less than 1-year of follow-up (N=8,355) | Reference | 0.71(0.60-0.83) | 0.54(0.45-0.64) | 0.43(0.35-0.52) |
| Using the complete dataset without imputation (N=12,043) | Reference | 0.70(0.63-0.79) | 0.56(0.49-0.63) | 0.53(0.46-0.60) |
| Competing risk model | Reference | 0.69(0.62-0.77) | 0.57(0.51-0.64) | 0.53(0.47-0.61) |

Notes:a: adjusted for age; sex; CCI, UA, TCho, Alb, eGFR, Hdl-c, TG, Ldl-c and Hb values; and the use of antidiabetic agents, antihypertensive agents, and statin agents.

**Table 6.** C-statistic, net reclassification improvement (NRI), and integrated discrimination improvement (IDI) for 1-year CVD and CKD progression after adding the log-transformed serum Cr/CysC ratio to the basic model

| **Models** | **C-statistics (95% CI)** | **Model performance compared to baseline model** | | |
| --- | --- | --- | --- | --- |
|  |  | **ΔC-index (95% CI)** | **NRI (95% CI)** | **IDI (95% CI)** |
| **Cardiovascular Disease** |  |  |  |  |
| Model1 | 0.809(0.799-0.818) | - | - | - |
| Model2 | 0.810(0.800-0.819) | 0.001(0.001-0.002) | 0.014(-0.011-0.026) | 0.001(0.0001-0.002) |
| **Chronic kidney disease Progression** |  |  |  |  |
| Model1 | 0.689(0.679-0.698) | - | - | - |
| Model2 | 0.697(0.687-0.706) | 0.008(0.007-0.008) | 0.023(0.008-0.053) | 0.008(0.005-0.011) |

Notes:Model1: adjusted for age; sex; CCI, UA, TCho, Alb, eGFR, Hdl-c, TG, Ldl-c and Hb values; and the use of antidiabetic agents, antihypertensive agents, and statin agents.

Model2: adjusted for Model1+log creatinine-cystatin C ratio.

**Figure 1.** Box plots showing the distribution of the serum Cr/CysC ratio before and after log-transformation.


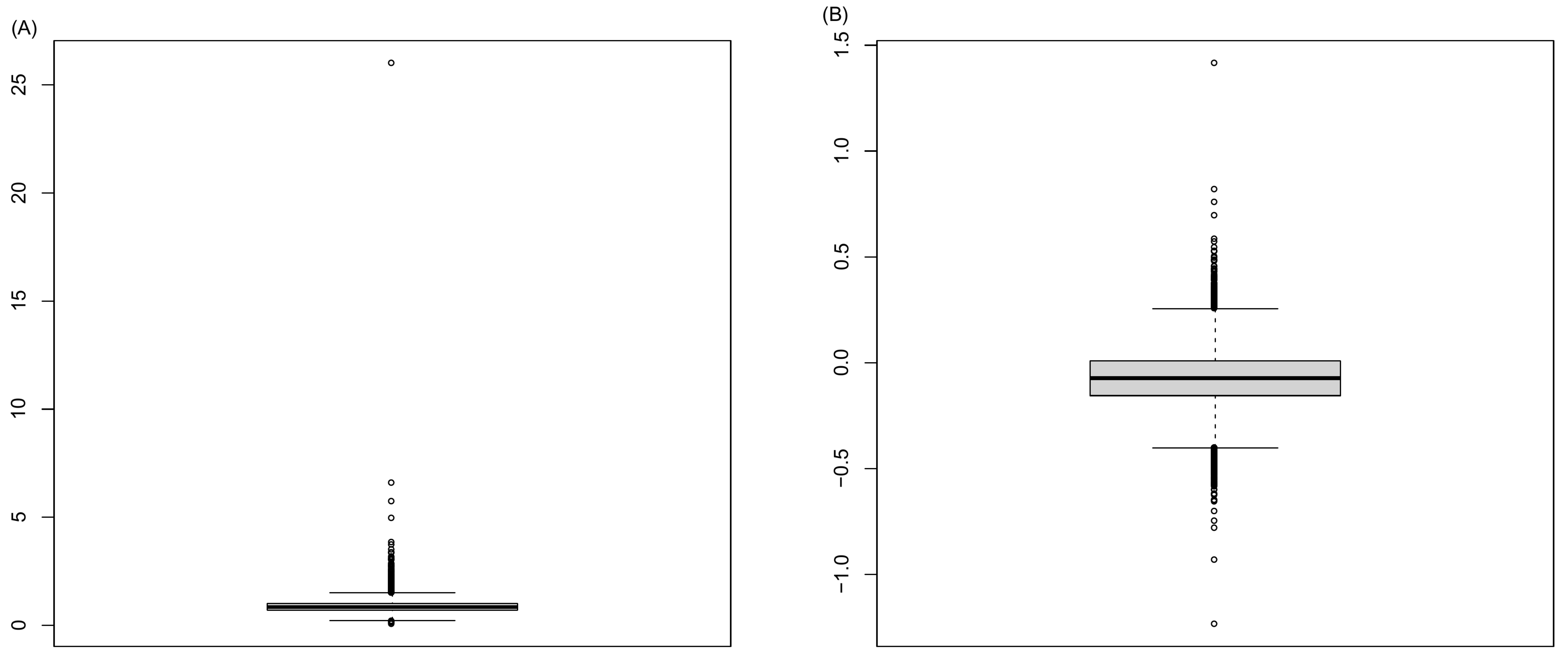


Notes:Box plots showing the distribution of A for the serum Cr/CysC ratio; B for the log-transformation serum Cr/CysC ratio.

**Figure 2.** Cubic spline model shows relationship of log-transformed serum Cr/CysC ratio with outcomes.


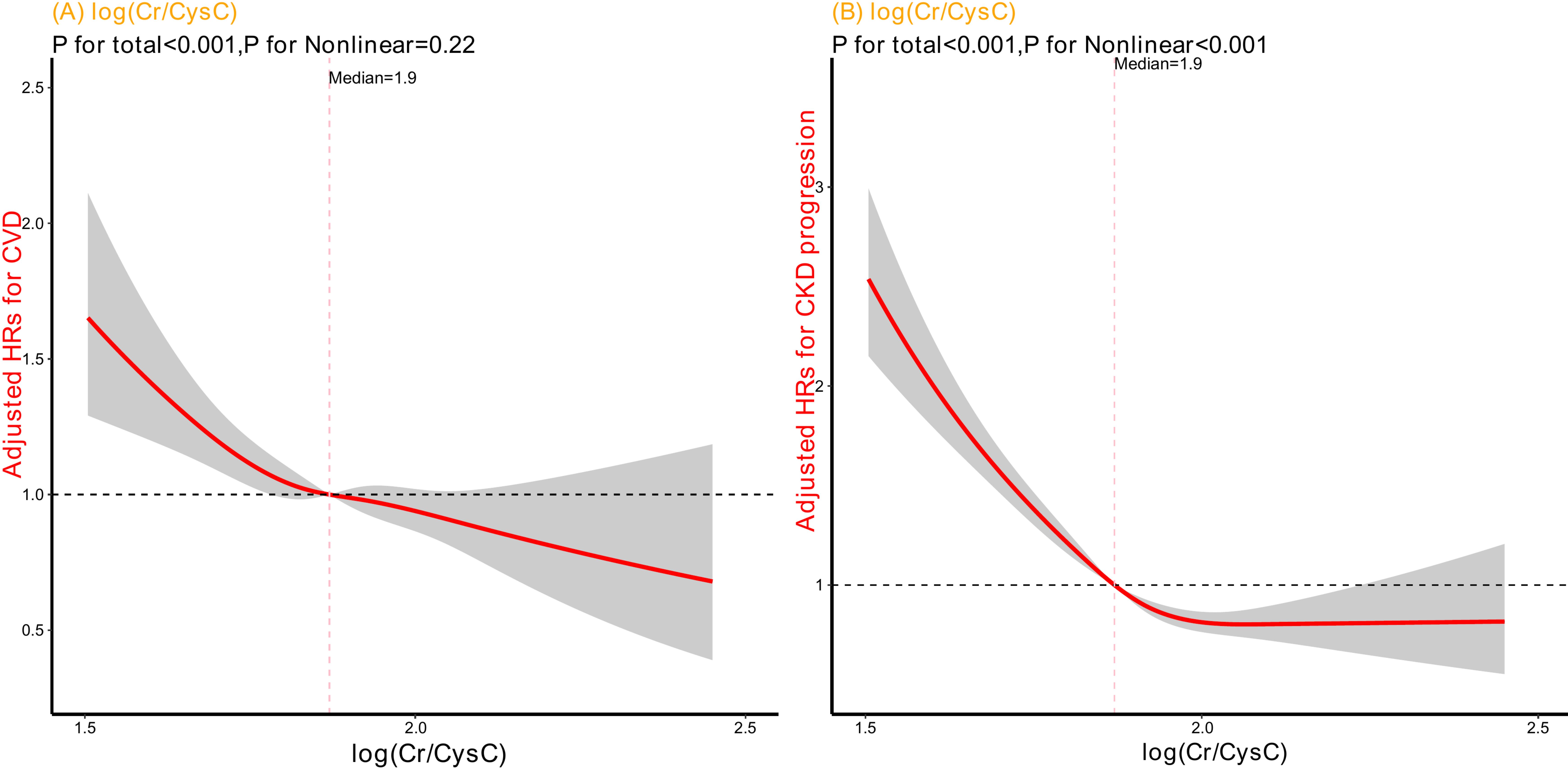


Notes: Cubic spline model shows relationship of log-transformation serum Cr/CysC ratio with (A) CVD events and (B) CKD progression. Model adjusted for adjusted for age; sex; CCI, UA, TCho, Alb, eGFR, Hdl-c, TG, Ldl-c and Hb values; and the use of antidiabetic agents, antihypertensive agents, and statin agents.
